# Sagliker Syndrome in a Patient with Secondary Hyperparathyroidism and Chronic Renal Insufficiency: A Case Report

**DOI:** 10.22038/aojnmb.2018.10567

**Published:** 2018

**Authors:** Sara Shakeri, Soroush Zarehparvar Moghadam, Ramin Sadeghi, Narjess Ayati

**Affiliations:** Nuclear Medicine Research Centre, Mashhad University of Medical Sciences, Mashhad, Iran

**Keywords:** Chronic renal failure, Renal osteodystrophy, Sagliker syndrome, Secondary hyperparathyroidism Skeletal deformity

## Abstract

Sagliker syndrome is a rare form of renal osteodystrophy resulted from untreated secondary hyperparathyroidism. It is described by severe skeletal deformities, high level of PTH in patients with chronic renal failure, and deformed face. This paper reports a 44-year-old male patient with the mentioned characteristics. In addition to the unique clinical features, high levels of ALP and PTH hormones encouraged us to search for syndrome-like a disease, which clinically and paraclinically matched the Sagliker syndrome.

This case highlights the importance of clinicians’ attention for early monitoring and appropriate treatment as it is shown to be effective in preventing irreversible complications such as soft tissue and bone abnormalities and cardiovascular impairment in patients with Sagliker syndrome. Therefore, considering the syndrome is recommended as one of the diagnostic hypothesis in young patients with renal insufficiency, secondary hyperparathyroidism, and skeletal deformities.

## Introduction

Sagliker syndrome (SS) was first described in 2004 by Sagliker et al. as a combination of secondary hyperparathyroidism and chronic renal insufficiency. This syndrome was noticed by finding two cases of chronic kidney disease (CKD) along with face deformity symptoms (1). The majority of patients with SS syndrome are young aged women (18-39 years) characterized by severe facial and skeletal changes, such as short stature, maxillary or mandibular destruction and fingertip deformities (2). In addition, secondary hyperparathyroidism (SHPT), especially when occurred in young ages, results in changes of musculoskeletal and other organs such as immune and cardiovascular systems (3). 

Moreover, it is proven that hyperplasia and hypertrophy of the parathyroid gland cells are related to the chronic renal failure, which results in the progressive production of parathyroid hormone (PTH). Following PTH rising, phosphorus clearance is decreased, resulting in hyperphosphatemia, which is the main cause of SHPT (3). Therefore, despite the low prevalence (approximately 0.5%) of SS syndrome among patients with chronic renal failure, it should be considered in the differential diagnosis of renal osteodystrophy (4). Even though renal transplantation stops musculoskeletal change progression, established deformities due to the SS are not reversible and affects the patient’ quality of life. Consequently, monitoring of SS in young aged patients with CKD seems to be appropriate (5). 

In this paper, we report a case of SS referred to our nuclear medicine department to perform a Tc99m-sestamibi scan. 

## Case report

**Figure 1:**
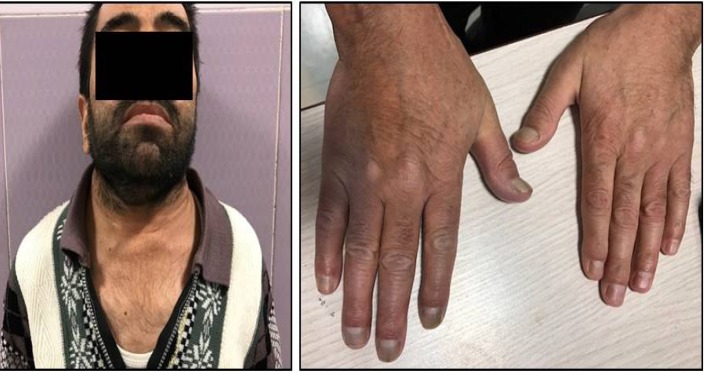
Patient’s appearance: Short stature, maxillary deformity, short neck, barrel chest, and fingertip deformity

A 44-year-old male patient referred to our center for the parathyroid scan. The patient had a history of renal insufficiency since 1999. Despite renal transplantation, he re-experienced renal failure after 5 years. In addition to SHPT and CKD, the patient suffered from severe maxillary and mandibular deformities, dental abnormalities, and a prominent forehead. Short neck and short stature were significant, as well as the barrel like chest (Figure 1).

Moreover, subtotal parathyroidectomy was performed for the patient in 2008. The patient had experienced two episodes of hypocalcemia along with weakness, dyspnea and neck bulging in the past 6 months.

^99m^Tc-sestamibi dual phase protocol was used for this purpose. Immediately after IV injection of 740 MBq (20 mCi) of ^99m^Tc-sestamibi, patients underwent early imaging of the neck and mediastinum using a dual-head E-CAM SPECT camera equipped with low-energy and high-resolution collimator. Immediately after early ^99m^Tc-sestamibi imaging (10 minutes post-injection), SPECT was performed. SPECT images (128×128 matrix using 64 projections over 360^0^ with 20 seconds per step). SPECT images were reconstructed by an iterative method (OSEM, number of iterations 8 subsets 4). One hundred and eighty min after injection delayed SPECT imaging of the neck was repeated and data were acquired and processed with the same protocol.

**Figure 2:**
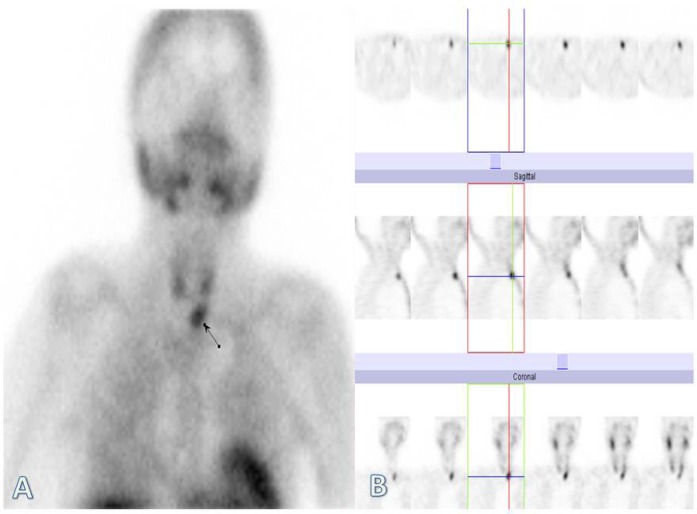
Tc-99m sestamibi scintigraphy of the parathyroid. The early phase images on planar (A) and SPECT views (B), showed radiotracer uptake in the thyroid region, as well as a focal zone of increased tracer uptake in the left lower part of the neck inferior to the thyroid

**Figure 3:**
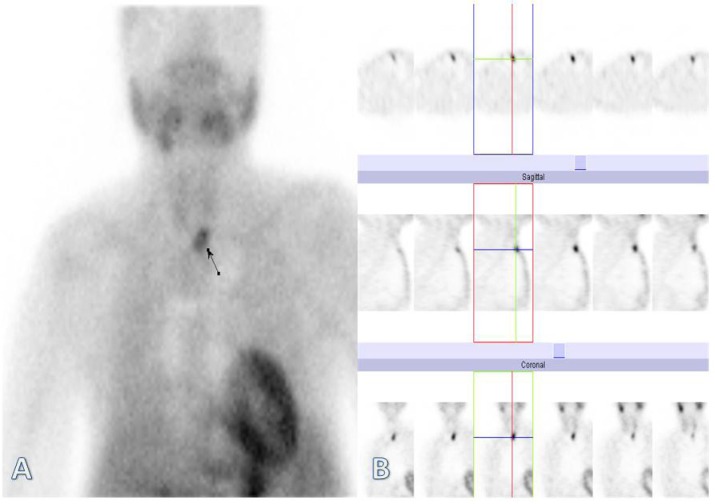
Tc-99m sestamibi scintigraphy of the parathyroid. The delayed phase images on planar (A) and SPECT views (B), showed tracer washout from the neck region with a focal area of retained activity in the left lower part of the neck inferior to the thyroid

The scan pattern on early phase images showed radiotracer uptake in the thyroid region, as well as a focal zone of increased tracer uptake in the left lower part of the neck inferior to the thyroid (Figure 2). Delayed images revealed tracer washout from the neck region with a focal area of retained activity in the left lower part of the neck inferior to the thyroid. The SPECT images also proved the above findings (Figure 3).

The patient underwent another parathyroidectomy and an abnormal parathyroid tissue was resected from the same location shown in our scan. PTH levels declined to a normal level postoperatively.

## Discussion

SS is a rare and severe form of renal insufficiency, which manifests with severe skeletal deformities caused by untreated SHPT (6). Different studies reported physical diagnostic criteria including maxillary, mandibular and dental deformities, skeletal changes like short stature, knee and scapula deformities, benign epithelial hyperplasia, fingertip changes, neurological and psychiatric disorders (1, 4, and 6).

It is shown that high levels of alkaline phosphatase and parathyroid hormone in patients with secondary hyperparathyroidism, who suffered from CKD for a long time, play a major role in SS induction (7). 

Although several studies have suggested the involvement of different factors in this syndrome, the exact cause is still unknown. Some genetic mutations were detected among 40% of patients with SS, which is possible to be associated with the pathogenesis of the syndrome (3 and 8-9). Tunç et al. suggested a correlation between Chromosome Aberrations (CAs) and the progression of SS (9). Moreover, it is shown that missense mutations on the GNAS1 gene play an important role in SS pathogenesis (8).

Since parathyroidectomy seems to be the most effective way in the treatment of SS by SHPT (2), SS is considered as a current indication of parathyroidectomy (6 and 10). In the presence of SS, total parathyroidectomy is preferred to avoid persistent and recurrence of the disease (10). 

However, total parathyroidectomy is the only definite way for stopping the progression of the disease, it should be noted that parathyroidectomy could not reverse the skeletal changes (3 and 11).
